# CBL0137 impairs homologous recombination repair and sensitizes high-grade serous ovarian carcinoma to PARP inhibitors

**DOI:** 10.1186/s13046-022-02570-4

**Published:** 2022-12-21

**Authors:** Xue Lu, Yaowu He, Rebecca L. Johnston, Devathri Nanayakarra, Sivanandhini Sankarasubramanian, J. Alejandro Lopez, Michael Friedlander, Murugan Kalimutho, John D. Hooper, Prahlad V. Raninga, Kum Kum Khanna

**Affiliations:** 1 QIMR Berghofer Medical Research Institute, 300 Herston Road, Herston, Brisbane, QLD 4006 Australia; 2 School of Environment and Sciences, Griffith University, Nathan, QLD 4111 Australia; 3 Mater Research Institute, The University of Queensland, Translational Research Institute, Woolloongabba, QLD 4102 Australia; 4 University of New South Wales Clinical School, Prince of Wales Hospital, Randwick, NSW 2031 Australia

**Keywords:** High-grade serous ovarian carcinomas, SSRP1, PARP inhibitor, CBL0137, Homologous recombination

## Abstract

**Background:**

High-grade serous ovarian carcinomas (HGSCs) are a heterogeneous subtype of epithelial ovarian cancers and include serous cancers arising in the fallopian tube and peritoneum. These cancers are now subdivided into homologous recombination repair (HR)-deficient and proficient subgroups as this classification impacts on management and prognosis. PARP inhibitors (PARPi) have shown significant clinical efficacy, particularly as maintenance therapy following response to platinum-based chemotherapy in BRCA-mutant or homologous recombination (HR)-deficient HGSCs in both the 1st and 2nd line settings. However, PARPi have limited clinical benefit in HR-proficient HGSCs which make up almost 50% of HGSC and improving outcomes in these patients is now a high priority due to the poor prognosis with ineffectiveness of the current standard of care. There are a number of potential lines of investigation including efforts in sensitizing HR-proficient tumors to PARPi. Herein, we aimed to develop a novel combination therapy by targeting SSRP1 using a small molecule inhibitor CBL0137 with PARPi in HR-proficient HGSCs.

**Experimental design:**

We tested anti-cancer activity of CBL0137 monotherapy using a panel of HGSC cell lines and patient-derived tumor cells in vitro. RNA sequencing was used to map global transcriptomic changes in CBL0137-treated patient-derived HR-proficient HGSC cells. We tested efficacy of CBL0137 in combination with PARPi using HGSC cell lines and patient-derived tumor cells in vitro and in vivo.

**Results:**

We show that SSRP1 inhibition using a small molecule, CBL0137, that traps SSRP1 onto chromatin, exerts a significant anti-growth activity in vitro against HGSC cell lines and patient-derived tumor cells, and also reduces tumor burden in vivo. CBL0137 induced DNA repair deficiency via inhibition of the HR repair pathway and sensitized SSRP1-high HR-proficient HGSC cell lines and patient-derived tumor cells/xenografts to the PARPi, Olaparib in vitro and in vivo. CBL0137 also enhanced the efficacy of DNA damaging platinum-based chemotherapy in HGSC patient-derived xenografts.

**Conclusion:**

Our findings strongly suggest that combination of CBL0137 and PARP inhibition represents a novel therapeutic strategy for HR-proficient HGSCs that express high levels of SSRP1 and should be investigated in the clinic.

**Supplementary Information:**

The online version contains supplementary material available at 10.1186/s13046-022-02570-4.

## Translational relevance

High-grade serous ovarian carcinomas (HGSCs) can be subdivided into homologous recombination (HR)-deficient and proficient subtypes. Maintenance therapy with PARP inhibitors (PARPi) following response to 1st line chemotherapy significantly increases progression free and overall survival in BRCA-mutant or HR-deficient HGSCs and has been a major advance. However, PARPi are not effective in HR-proficient HGSCs. Hence, developing a novel treatment strategy to broaden the use of PARPi in HR-proficient HGSC patients is clinically important. SSRP1, a component of the FAcilitates Chromatin Transcription (FACT) complex, is upregulated in ovarian carcinomas compared to normal tissue and is involved in regulation of tumor growth and DNA repair. We found that CBL0137, an SSRP1 inhibitor, inhibits the growth of HGSC cell lines and patient-derived tumor cells in vitro and in vivo. Furthermore, CBL0137 sensitized HR-proficient HGSC cells lines and patient-derived tumor cells/xenografts to PARPi by inhibiting HR repair of DNA damage. This combination strategy warrants clinical translation with investigation in patients with HR-proficient HGSCs who have a high unmet need.

## Introduction

Epithelial ovarian cancer (EOC) is the eighth most common cause of female cancer worldwide and is the most lethal gynecologic malignancy [1, 2]. Most EOCs are of the high-grade serous carcinoma (HGSC) histotype with poor survival rates due to late diagnosis [3, 4]. The standard of care for patients with HGSC includes surgical debulking with adjuvant or neoadjuvant platinum-based chemotherapy followed by maintenance therapy with a PARP inhibitor in patients with *BRCA* pathogenic mutations as well as those with homologous recombination (HR) defects [5].

Approximately 50% of HGSCs have dysfunctional DNA repair via defects in the HR pathway [3]. The synthetic lethality between HR-deficiency (HRD) and poly (ADP-ribose) polymerase (PARP) inhibition established a significant efficacy of PARP inhibitors (PARPi) in *BRCA1/2* mutated or HR-deficient HGSC patients [6]. Currently, Olaparib, Rucaparib and Niraparib are FDA-approved PARPi for HGSC patients [7]. Recent phase 3 trials demonstrated clinically meaningful improvement in overall survival with maintenance therapy with a PARPi particularly in patients with pathogenic variants in *BRCA* and/or *BRCA* wild-type HRD HGSC. PARPi are now considered standard of care in patients with HRD [8–10]. However, PARPi have shown limited to no clinical benefit in HR-proficient HGSC patients. These make up almost 50% of patients with HGSC and highlights the need for novel therapeutic strategies for this large number of patients with advanced HGSC.

SSRP1, a component of the FAcilitates Chromatin Transcription (FACT) complex, is a histone chaperone and has a well characterized role in assisting RNA Pol-II during transcription elongation [11]. SSRP1 regulates multiple key modulators of cancer progression such as MYC, NF-ĸβ, and p53, and contributes critically to key cellular processes including DNA replication, DNA repair, cell cycle and transcription [11–13]. In particular, SSRP1 plays an important role in DNA damage repair through chromatin remodeling and interacting with the proteins PARP and XRCC1 [14]. SSRP1 also facilitates H2A.X dependent signaling of DNA damage repair via promoting deposition of newly synthesized H2A.X at DNA damage sites [15]. Additionally, SSRP1 is associated with HR pathways via physically interacting with the key HR regulatory protein RAD54 [16]. SSRP1 is upregulated in multiple cancers especially in poorly differentiated tumors including breast cancer, non-small-cell lung cancer, colorectal adenocarcinoma, and pancreatic ductal carcinomas [12, 17], and its protein expression has been shown to increase in ovarian cancer cell lines and patient tumor tissues compared to the normal cells or tissues [18]. High SSRP1 expression is seen in both early (57%; stage II) and late-stage tumors (95%; stage III and IV) [18]. This supports SSRP1 as a potential therapeutic target in HGSC and given its role in DNA repair, its inhibition may also promote HRD and hence sensitize HGSCs to PARPi.

CBL0137, the FACT complex inhibitor, is a small molecule curaxin (carbazole-based) compound. The mechanism of action of CBL0137 is mainly through DNA intercalation and disturbing the DNA-histone interactions within nucleosomes (19,20). CBL0137 is also known to target the 3D spatial genome organization and induce disruption of enhancer-regulated transcription [19]. Moreover, CBL0137 acts on FACT by chromatin trapping, wherein FACT is redistributed from the nucleoplasm and is tightly bound to different components of disassembled chromatin [20, 21]. Such chromatin-trapping further affects the downstream cellular processes mediated by FACT, such as p53 activation, NOTCH1 suppression and NF-κβ inhibition, and thus inhibits tumor progression [22–24]. CBL0137 has been shown to effectively inhibit tumor proliferation in several cancers, including glioblastoma, renal cell carcinoma, melanoma, neuroblastoma, and small cell lung cancer [24–28]. Currently, CBL0137 is being tested in multiple phase 1/2 clinical trials in patients with hematological malignancies (NCT02931110), extremity melanoma or sarcoma (NCT03727789), and glioblastoma (NCT01905228).

In the present study, we investigated the therapeutic effects of CBL0137 monotherapy on the growth of HGSC cell lines and patient-derived tumor cells in vitro and in vivo. We also provide an innovative therapeutic approach by targeting SSRP1 using CBL0137 to exert a synergistic anti-cancer activity with PARPi in *BRCA1/2* wild-type and HR-proficient HGSC cells by impairing DNA repair via targeting HR pathway.

## Methods

### Cell lines and reagents

HGSC cell lines including OVCAR-8, OVCAR-4, OVCAR-3, Kuramochi, CAOV3, OV90, and OAW42 and non-cancerous ovarian epithelial cell lines HOSE6.3 and HOSE17.1 were kindly provided by Dr. Elaine Sanij (St Vincent’s Hospital, QLD). PEO1 and PEO4 cell lines were provided by Prof John Hooper. BRCA1/2 gene mutation status and HR status of HGSC established cell lines is given in Additional file 3 Table S1. All HGSC cell lines were cultured in RPMI1640 media containing 10% fetal bovine serum (FBS) (Gibco). Murine ID8 (both ID8 p53^−/−^ and ID8 p53^−/−^ BRCA1^−/−^) cells [29] were kindly provided by Prof Iain McNeish at Imperial College of London. All cell lines were tested for Mycoplasma infection at QIMR Berghofer Medical Research Institute. CBL0137 (Cat #: S8483), Olaparib (Cat #: S1060) and Rucaparib (Cat #: S4948) were purchased from Selleck Chemicals.

### Patient-derived tumor cells and xenografts

Ascites-derived HGSC patient-derived xenograft (PDX) models including DF20, DF68, DF86, DF101, DF106, DF149, and DF181 were obtained from the Dana-Farber Cancer Institute, USA [30]. BRCA1/2 gene mutation status and HR status of HGSC patient-derived tumour cells is given in Additional file 3 Table S1. All of these PDXs were expanded and passaged into 6-weeks old female NRG mice (NOD/RAG1/2^−/−^/IL2Rγ^−/−^) mice, and stored for future use in Bambanker frozen medium at − 80 °C for in vivo and in vitro studies. Tumor cells were used to generate patient-derived cell lines for experiments in vitro, and were maintained in RPMI-1640 medium containing 10% FBS for short-term culture, at 37 °C with 5% CO_2_. HGSC PDX model LP28 was established from a chemotherapy-treated HGSC patient and was established and provided by Prof John Hooper.

### Protein fractionation

Protein fractions of OVCAR-8 cells after CBL0137 (0-2.5 μM) treatment for cytoplasmic and chromatin-bound fractions were generated as described previously [31]. Briefly, OVCAR-8 cells were harvested and resuspended in lysis buffer [10 mM HEPES, 10 mM KCl, 0.05% NP-40, protease inhibitor cocktail (Sigma), and phosphatase inhibitor (Roche)] for 20 mins on ice. Lysates were centrifuged and cytoplasmic protein was collected as the supernatant. Pellets were then incubated with low salt buffer (10 mM Tris-HCl, 0.2 mM MgCl_2_, 1% Triton X-100, protease inhibitor cocktail, and phosphatase inhibitor) for 15 mins on ice. The samples were centrifuged at 14,000 rpm for 10 mins and nuclear proteins were obtained from the supernatant. The remaining pellets were lysed in 7 M urea buffer and the chromatin fraction was collected after sonication. Fractions obtained from fresh cell pellets were kept at − 20 °C until western blot analysis.

### RNA sequencing (RNA-seq)

DF149 cells, cultured in vitro for approximately 3-5 passages, were used for RNA-seq experiments. DF149 cells were collected after 8 h treatment with vehicle control (DMSO) or CBL0137 (2.5 μM). Three biological replicates per condition were obtained with cells cultured from 3 serial passages. Total RNA was extracted using the RNeasy Plus Mini Kit (Qiagen) per manufacturer’s instruction. Strand-specific RNA-seq was performed by BGI Genomics using the polyA-selection method. Libraries were constructed and sequenced on the G-400 platform (BGI Group) with 2 × 100 bp reads sequenced to a minimum of 20 million read pairs per sample. Sequence reads were trimmed for adapter sequences using Cutadapt version 1.9 [32] and aligned using STAR version 2.5.2a [33] to the GRCh37 assembly with the Ensembl release 70 gene annotation. Quality control metrics were computed using RNA-SeQC version 1.1.8 [34] and transcripts were quantified using RSEM version 1.2.30 [35]. All downstream RNA-seq analysis was performed using R version 3.6.2 [36]. Differential expression analysis was performed using edgeR’s quasi-likelihood pipeline version 3.28.0 [37–39]. Specifically, only protein-coding genes that obtained sufficiently large counts were kept for further analysis, as determined by edgeR’s filter By Expr function with default settings. The design matrix was defined using model.matrix (~ 0 + Group), where Group is a factor indicating Treated and Control samples. The glmTreat function was used to conduct gene wise statistical tests for the contrast “Treated – Control” relative to a log2-fold-change threshold of log2 (1.5). *P*-values were adjusted for multiple testing using the R function p.adjust and method “fdr”. Differentially expressed genes were determined using a false discovery rate < 0.05. To perform pathway analysis, gene IDs were converted from Ensembl to Entrez using the bitr function from cluster Profiler version 3.14.3 [40], then passed to the enrich Pathway function from Reactome PA version 1.30.0 [41], before plotting the results with the Dotplot function from cluster Profiler. Heat maps were produced using the Heatmap function from Complex Heatmap version 2.2.0 [42] using scaled and centered log2 CPM values as input, with default row distance (“euclidean”) and clustering (“complete”) methods. Gene set enrichment analysis was performed using GSEA for desktop version 4.1.0. The expression data file (Gene Cluster Text, GCT) and phenotype data file (Categorical Class, CLS) were created in R as per the GSEA User Manual. The values from edgeR’s cpm function with defaults were used as the basis of the normalized expression GCT file. After loading the GCT and CLS files into GSEA, a standard GSEA analysis was performed using the following parameters: gene sets database “Hallmarks” (h.all.v7.5.symbols.gmt), permutation type “gene set”, seed for permutation value of 42, and the remaining parameters kept as default.

### HR reporter assay

The HR reporter assay was performed as described previously [43]. Briefly, OVCAR-8 cells were transfected with 1 μg pDR-GFP, a non-expressing eGFP plasmid, with 0.75 μg pCBASceI, a vector that expresses I-SceI nuclease. After 24 h, the transfected cells were treated with 0-2.5 μM of CBL0137 for further 24 h. Cells were then harvested and analyzed on a BD FACS Canto II flow cytometry. Flow cytometry analysis was performed using FlowJo V 10 (Tree Star, Ashland, Oregon, USA).

### Immunofluorescence

HGSC cells were seeded and incubated on coverslips overnight. Then the cells were treated with CBL0137 (0 or 1 μM) for 3 h prior to 6-Gy gamma irradiation challenge. For RPA32 foci study, cells were treated with 0 or 1 μM of CBL0137 for 6 h. Cells were fixed in 4% paraformaldehyde in PBS for 6 h after irradiation, then processed for immunofluorescence as described previously [44]. Antibodies used for immunofluorescence were against γ-H2A.X (Abcam Cat #: ab11174, 1:3000), RAD51 (GeneTex Cat #: GTX70230, 1:500), pRPA32 (S4/S8) (Bethyl Laboratories, Cat #: A300-245A, 1:1000), and DAPI (Sigma-Aldrich Cat #: D9564, 1:500). Images were acquired with a Delta Vision Deconvolution microscope and analysed with ImageJ software. A minimum of 100 cells per sample were analysed.

### DNA fiber assay

OVCAR-8 and OVCAR-4 cells were pre-treated with 1 μM CBL0137 for 3 h, followed by sequential pulse-labelling with 100 μM 5-Chloro-2′-deoxyuridine (CldU) (Sigma Cat #C6891) and 100 μM 5-Iodo-2′-deoxyuridine (IdU) (Sigma Cat #: I7125) for 25 mins each. Cells were then washed and harvested in 1xPBS. Cell lysis, DNA extraction, spreading, denaturation, and immunostaining were then performed as described previously [45]. Anti-BrdU (Abcam, Cat #: ab6326, 1:300), and anti-BrdU (BD Biosciences, Cat #: 347583, 1:75) were applied. Slides were visualized using Delta Vision Deconvolution microscope and analysed with ImageJ software.

### *In vivo* xenografts

All experiments were conducted in accordance with the protocols approved by QIMR Berghofer Medical Research Institute Animal Ethics Committee and the University of Queensland Animal Ethics Committee. For syngeneic murine ID8 (*p53*^*−/−*^*, BRCA1*^*−/−*^) HGSC model, 3 × 10^6^ cells were prepared in 50% growth factor reduced Matrigel (BD, Biosciences, Bedford, USA)/PBS and injected in 6-weeks old female C57BL/6 mice via intraperitoneal injections. Two HGSC PDX models, DF149 (*BRCA1/2* wild-type and HR-proficient) and DF86 (*BRCA1/2* mutant and HR-deficient), were used to test CBL0137 treatment in vivo. Luciferase-tagged DF149 and DF86 cells were injected intraperitoneally in 6-weeks old female NRG mice. The tumor growth was monitored by bioluminescent imaging using a Xenogen IVIS Spectrum (Caliper life sciences). For testing CBL0137 single agent activity, mice were randomized into two treatment groups once tumors were established. Mice were then treated with either vehicle or CBL0137 (60 mg/kg, IV, once per week) for 2 weeks. For ID8 (*p53*^*−/−*^*, BRCA1*^*−/−*^) models, at the end of two-week treatment we collected and measured the volume of ascitic fluid and also counted the number of tumor nodules within the intraperitoneal cavity of mice.

For CBL0137 and Olaparib combination treatments, mice were randomized into four treatment groups that consisted of vehicle, CBL0137 (30 mg/kg, i.v, once per week), Olaparib (50 mg/kg, i.p, Monday-Friday), and the combination of CBL0137 and Olaparib. Mice were treated for 2 weeks. For CBL0137 and Olaparib combination group, tumor-bearing mice were pre-treated with CBL0137 for 24 h prior to the treatment with Olaparib. Tumor growth was monitored at the end of treatment by bioluminescence imaging (IVIS). Kaplan-Meier survival analysis was used to analyze the effect of drug combinations on overall survival for ID8 and DF149 models.

For CBL0137 and carboplatin combination therapy, LP28 HGSC PDX, tumor slurry was injected into the intraperitoneal cavity of 6-weeks old female NSG mice. Once tumor became palpable in the intraperitoneal cavity, mice were randomized into four treatment groups consisting of vehicle control, CBL0137 (30 mg/kg, i.v, once per week), carboplatin (2 mg/kg, i.p, twice per week), and the combination of CBL0137 and Olaparib, with 8 mice per treatment group. Mice were then treated for 2 weeks, and survival analysis was performed using the Kaplan-Meier survival analysis.

### Statistical analysis

All values are presented as mean ± SEM unless specified. Data were analyzed using GraphPad Prism 8 (GraphPad Software, USA). Survival curves were plotted by Kaplan-Meier survival analysis using GraphPad Prism 8 and evaluated with log-rank tests. Statistical significance was determined by Unpaired Student’s t-tests, One-way ANOVA, or Two-way ANOVA and the *P* values ≤0.05 represent significant difference (* for *P* ≤ 0.05, ** for *P* ≤ 0.01, *** for *P* ≤ 0.001, and **** for *P* ≤ 0.0001).

## Results

### CBL0137 inhibits proliferation of HGSC cell lines and PDX lines with high SSRP1 expression

**Fig. 1:**
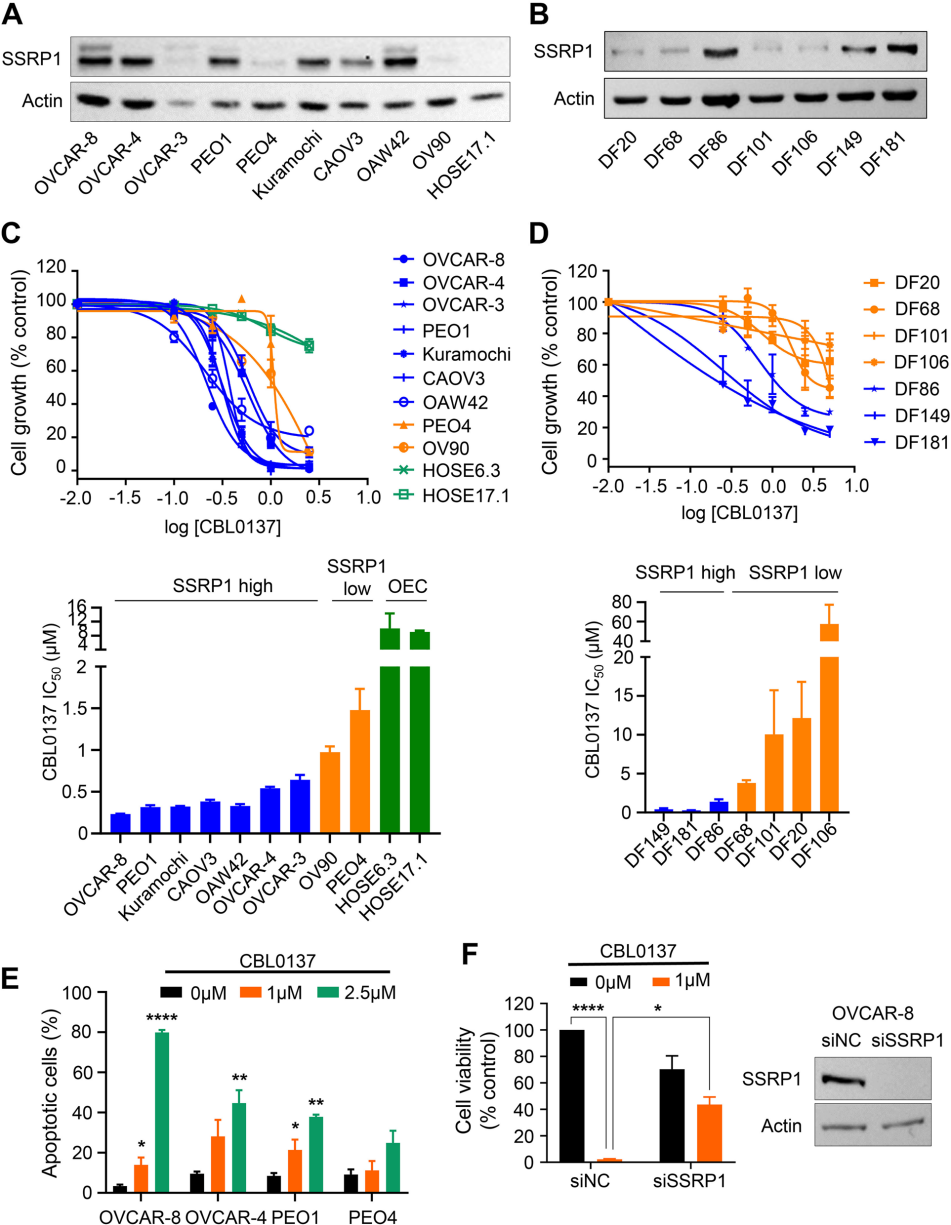
CBL0137 inhibits proliferation of HGSC cell lines and PDX lines. **A**, **B** SSRP1 protein expression was evaluated by Western blotting in the panel of HGSC cell lines (**A**) and patient-derived tumour cells (**B**). Actin was used as loading control. **C**, **D** A panel of HGSC cell lines (**C**) and patient-derived tumor cells (**D**) (blue – SSRP1-high, orange – SSRP1-low, and green – non-cancerous ovarian epithelial cells (OEC)) were treated with CBL0137 (0-2.5 μM) for 6 days. Cell viability was assessed using MTS assay (*n* = 3). The dose response curve was generated by GraphPad Prism 8. IC_50_ values of CBL0137 on a panel of HGSC cell lines (**C**) and patient-derived tumor cells (**D**). The data represented as mean IC_50_ value of three biological replicates. **E** OVCAR-8, OVCAR-4, PEO1, and PEO4 cells were treated with CBL0137 (0-2.5 μM) for 48 h and apoptosis was measured by Annexin V FITC/PI flow cytometry analysis. One-way ANOVA analysis was employed for statistical significance (*n* = 3). **F** OVCAR-8 cells were transfected with scramble (siNC) or SSRP1-specific siRNA for 24 h, followed by the treatment with 1 μM CBL0137 for 48 h. Left panel: Cell viability of OVCAR-8 cells, where unpaired Student’s t-tests were employed for statistical significance. Right panel: Representative Immunoblot for OVCAR-8 cells shows knockdown efficiency of SSRP1. Unpaired Student’s t-tests were employed for statistical significance. Representative Immunoblot shows knockdown efficiency of SSRP1

Since SSRP1 expression is significantly upregulated in ovarian cancer tissues compared to normal tissues [18], we first evaluated SSRP1 protein levels in a panel of 9 established HGSC cell lines, non-cancerous ovarian epithelial cell line HOSE17.1, and a panel of 7 HGSC patient-derived tumor cells. The majority of the HGSC lines expressed moderate to high SSRP1 protein levels, whereas PEO4 and OV90 cells expressed low levels of SSRP1 protein (Fig. 1A). Interestingly, no SSRP1 expression was detected in non-cancerous ovarian epithelial cell line HOSE17.1 (Fig. 1A). Also, three of seven patient-derived tumor lines including DF86, DF149, and DF181, expressed high levels of SSRP1 compared to the other four patient-derived tumor lines (Fig. 1B). Next, we examined the effect of SSRP1 knockdown on the viability of a panel of HGSC cell lines using a long-term colony formation assay. Our results demonstrated that SSRP1 downregulation significantly reduced the viability of SSRP1-high HGSC cell lines compared to SSRP1-low cell lines (Additional file 2, Fig. S1A, B), suggesting that SSRP1-high lines are dependent on its overexpression for survival.

We next evaluated the in vitro effects of CBL0137, a drug that traps SSRP1 into chromatin (Additional file 2, Fig. S1C), on the growth of HGSC cell lines and HGSC patient-derived tumor cells expressing varying levels of SSRP1. The IC_50_ values of CBL0137 were observed to be 0.2–0.6 μM in HGSC cells with moderate and high SSRP1 expression compared to 1.0–1.4 μM in HGSCs with lower SSRP1 expression (Fig. 1C). Similarly, The IC_50_ values of CBL0137 were 0.3–1.3 μM in SSRP1-high patient-derived tumor cells compared to > 3.8 μM in SSRP1-low patient-derived tumor cells (Fig. 1D). Interestingly, CBL0137 showed no significant growth inhibitory effect on non-cancerous ovarian epithelial cells HOSE17.1 and HOSE6.3 (Fig. 1C). Moreover, in SSRP1-high OVCAR-8, OVCAR-4, and PEO1 cells growth inhibitory effect of CBL0137 was associated with concentration-dependent induction of apoptosis measured by Annexin V staining and FACS analysis (Fig. 1E, Additional file 2 Fig. S2A). However, no significant increase in Annexin V positive apoptotic cells was observed upon CBL0137 treatment in SSRP1-low PEO4 cells (Fig. 1E, Additional file 2 Fig. S2A). Hence, our data suggested that induction of cell apoptosis contributes to the anti-proliferation activity of CBL0137. Moreover, CBL0137 treatment also resulted in a marked cleavage of a classical apoptosis marker, caspase-3, and its substrate PARP1, in SSRP1-high HGSC lines (OVCAR-8, OVCAR-4, and PEO1) cells, but not in SSRP1-low PEO4 cells (Additional file 2, Fig. S2B). Next, we investigated the expression of pro-apoptotic and anti-apoptotic proteins and found that CBL0137 treatment resulted in increased expression of pro-apoptotic BIM protein and downregulation of a classical anti-apoptosis protein, MCL1 in SSRP1-high HGSC lines but not in SSRP1-low HGSC cells indicating induction of apoptosis upon CBL0137 treatment (Additional file 2, Fig. S2B). These results indicated that CBL0137 induces caspase-3-dependent apoptosis in SSRP1-high HGSC cells. Notably, CBL0137-induced apoptosis was associated with significant accumulation of SSRP1-high HGSC (OVCAR-8, OVCAR-4, and PEO1) cells in the G2/M phase of the cell cycle (Additional file 2, Fig. S2C).

To further confirm if SSRP1 expression determines CBL0137 sensitivity in HGSC cells, we depleted SSRP1 expression using SSRP1-specific siRNAs followed by treatment with CBL0137. SSRP1 depletion significantly rescued OVCAR-8 cells from CBL0137-induced cell death (Fig. 1F), suggesting that SSRP1 expression determines CBL0137 response in HGSC cells. Taken together, our data indicates that CBL0137 selectively induces cell cycle arrest and apoptosis in SSRP1-high HGSC lines, suggesting that CBL0137 as a single agent may provide therapeutic benefit in HGSC tumors with elevated SSRP1 expression.

### CBL0137 reduces tumor burden in HGSC syngeneic and PDX models in vivo

**Fig. 2:**
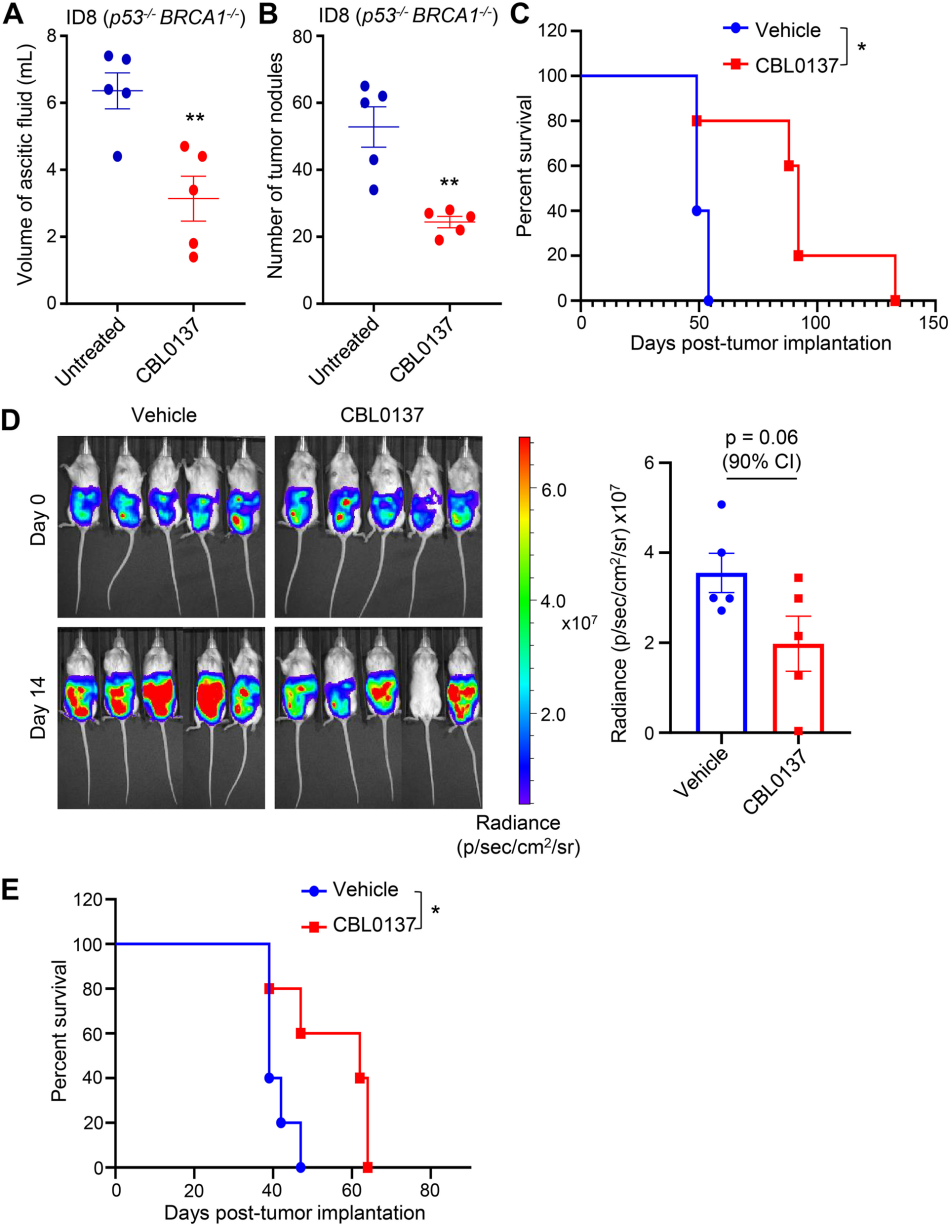
CBL0137 reduces tumor burden in HGSC syngeneic model and PDX model in vivo. **A**, **B** ID8 (p53^−/−^ BRCA1^−/−^) cells were engrafted intraperitoneally in 6-week-old female C57BL/6 mice and treated with either vehicle or CBL0137 (60 mg/kg, IV, once per week) for two weeks (*n* = 5). Effects of CBL0137 treatment on the volume of ascitic fluid (**A**) and on the number of tumor nodules (**B**) were analyzed. Unpaired Student’s t test was used (** for *P* ≤ 0.01). **C** Kaplan-Meier survival curves of ID8 (p53^−/−^ BRCA1^−/−^) syngeneic mouse models after CBL0137 treatment. Log-rank test was applied for statistical significance analysis (*P* = 0.021). **D** Luciferase-tagged DF86 tumor-bearing NRG mice were treated with vehicle or CBL0137 (60 mg/kg, i.v, once per week) (*n* = 5 mice in each group) for two weeks. Tumor growth was monitored by bioluminescence imaging (*n* = 5). **E** Kaplan-Meier survival of DF86 PDX after CBL0137 treatment. Log-rank test was applied for statistical significance analysis (*P* = 0.033)

We next investigated the anti-tumor activity of CBL0137 in vivo using the syngeneic ID8 (*p53*^*−/−*^ *BRCA1*^*−/−*^) HGSC model in immunocompetent mice [29]. CBL0137 treatment significantly reduced the volume of ascitic fluid (Fig. 2A) and also reduced the number of tumor nodules in the peritoneum of tumor-bearing mice (Fig. 2B). We also examined if CBL0137 treatment improves overall survival in mice engrafted with ID8 (*p53*^*−/−*^ *BRCA1*^*−/−*^) HGSC cells. CBL0137 treatment significantly prolonged the survival in mice where the median survival was extended to 92 days compared to the median survival of 49 days in vehicle-treated group (Fig. 2C). We next evaluated the anti-tumor activity of CBL0137 using PDX model in immunocompromised mice. We used luciferase-labelled DF86 PDX generated from a *BRCA1* and p53 mutant HGSC patient treated with 6 prior lines of chemotherapies [30]. DF86 cells express high levels of SSRP1 protein and are resistant to PARPi (Fig. 1B). Cohorts of NRG mice bearing DF86 HGSC tumors were treated with vehicle or CBL0137, and tumor growth was monitored by bioluminescence imaging. CBL0137 treatment significantly reduced tumor burden in DF86 tumor-bearing mice (Fig. 2D). Moreover, survival analysis revealed that CBL0137 treatment significantly prolonged the survival of DF86 tumor-bearing mice to 62 days compared to the median survival of 39 days in vehicle-treated mice (Fig. 2E). Overall, these observations demonstrate that CBL0137 treatment is effective as a monotherapy in vivo and prolongs median survival in HGSC models that have germline BRCA1 mutation and express high SSRP1 protein levels*.*

### CBL0137 impairs HR-mediated DNA repair in HGSC cells

**Fig. 3:**
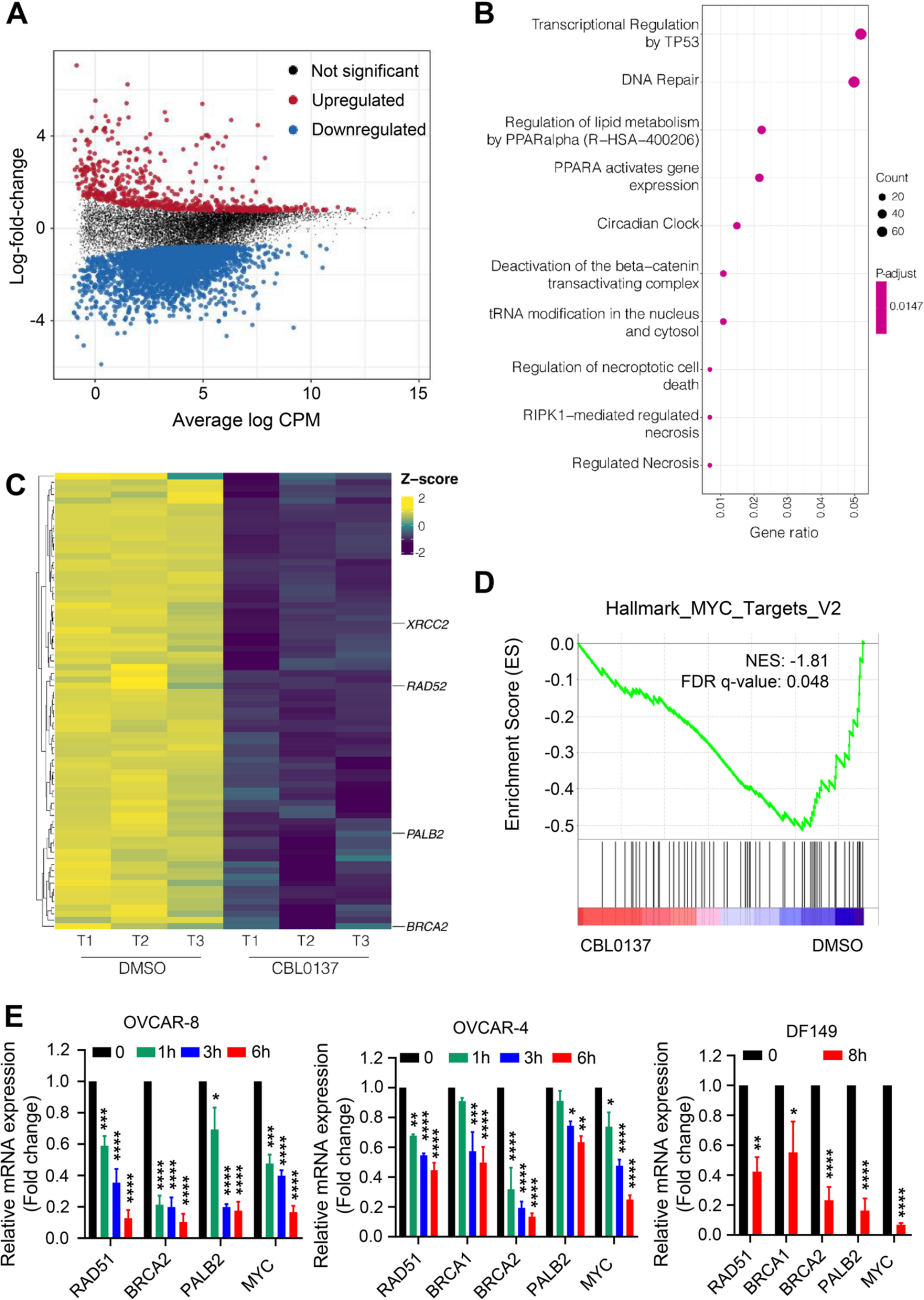
CBL0137 treatment downregulates HR genes in HGSCs. **A** Mean-difference plot for differential expression analysis between CBL0137-treated and vehicle control DF149 HGSC cells. Significantly upregulated and downregulated genes are colored red and blue, respectively. Significance based on FDR < 0.05. **B** Dot plot of the top 10 results from Reactome pathway analysis using the significantly downregulated genes (logFC < 0 and FDR < 0.05) in CBL0137-treated samples. **C** Heatmap of the significantly downregulated genes contributing to Reactome Pathway “DNA Repair” (R-HSA-73894). The genes with logFC < 0 and FDR < 0.05 from differential expression analysis of CBL0137-treated vs. vehicle control were used as input to Reactome. **D** Gene Set Enrichment Analysis showing MYC target V2 gene signature are enriched in DF149 cells upon DMSO treatment. **E** OVCAR-8, OVCAR-4, and DF149 cells were treated with 1 μM of CBL0137, for indicated time period. The mRNA expression of indicated genes was evaluated by RT-qPCR (*n* = 3). Statistical significance was determined by Unpaired Student’s t-tests

To decipher the molecular mechanism for anti-cancer activity of CBL0137 in SSRP1-high HGSC cells, whole transcriptome expression profiling (RNA-seq) of patient-derived HR-proficient DF149 tumor cells was performed in vitro after treatment with DMSO or CBL0137 (2.5 μM) for 8 h. Differential expression analysis using a minimum log2-fold-change threshold of log2 (1.5) identified 2818 significantly downregulated genes (logFC < 0, FDR < 0.05) and 638 significantly upregulated genes (logFC > 0, FDR < 0.05) in response to CBL0137 treatment (Fig. 3A, Additional file 3 - Supplementary Table S4). Reactome pathway analysis of the 2818 down-regulated genes revealed that the DNA repair pathway (R-HSA-73894) was enriched in CBL0137-treated cells compared to DMSO cells (Fig. 3B, Additional file 2 - Supplementary Table S5 and S6). Amongst the HR pathway genes that were most significantly downregulated in CBL0137-treated DF149 cells were *BRCA2*, *RAD52*, *XRCC2*, and *PALB2* (Fig. 3C). Additionally, Gene Set Enrichment Analysis (GSEA) revealed that the MYC targets V2 gene set was enriched in DMSO-treated DF149 cells, and thus negatively correlated with CBL0137-treated cells (Fig. 3D, Additional file 3 - Supplementary Table S7 and S8), where MYC is known to transcriptionally regulate the expression of HR pathway genes [46]. Downregulation of *MYC* and key HR-pathway genes including *BRCA1*, *BRCA2*, *RAD51*, and *PALB2* after treatment with CBL0137 was validated using RT-qPCR in two SSRP1-high HGSC lines (OVCAR-8, OVCAR-4) and patient-derived DF149 cells (Fig. 3E). CBL0137 treatment significantly decreased expression of these genes in a time-dependent manner. Taken together, our results demonstrated that CBL0137 treatment imparts BRCA-ness and possible HR-deficiency through downregulating expression of MYC and HR genes.

**Fig. 4:**
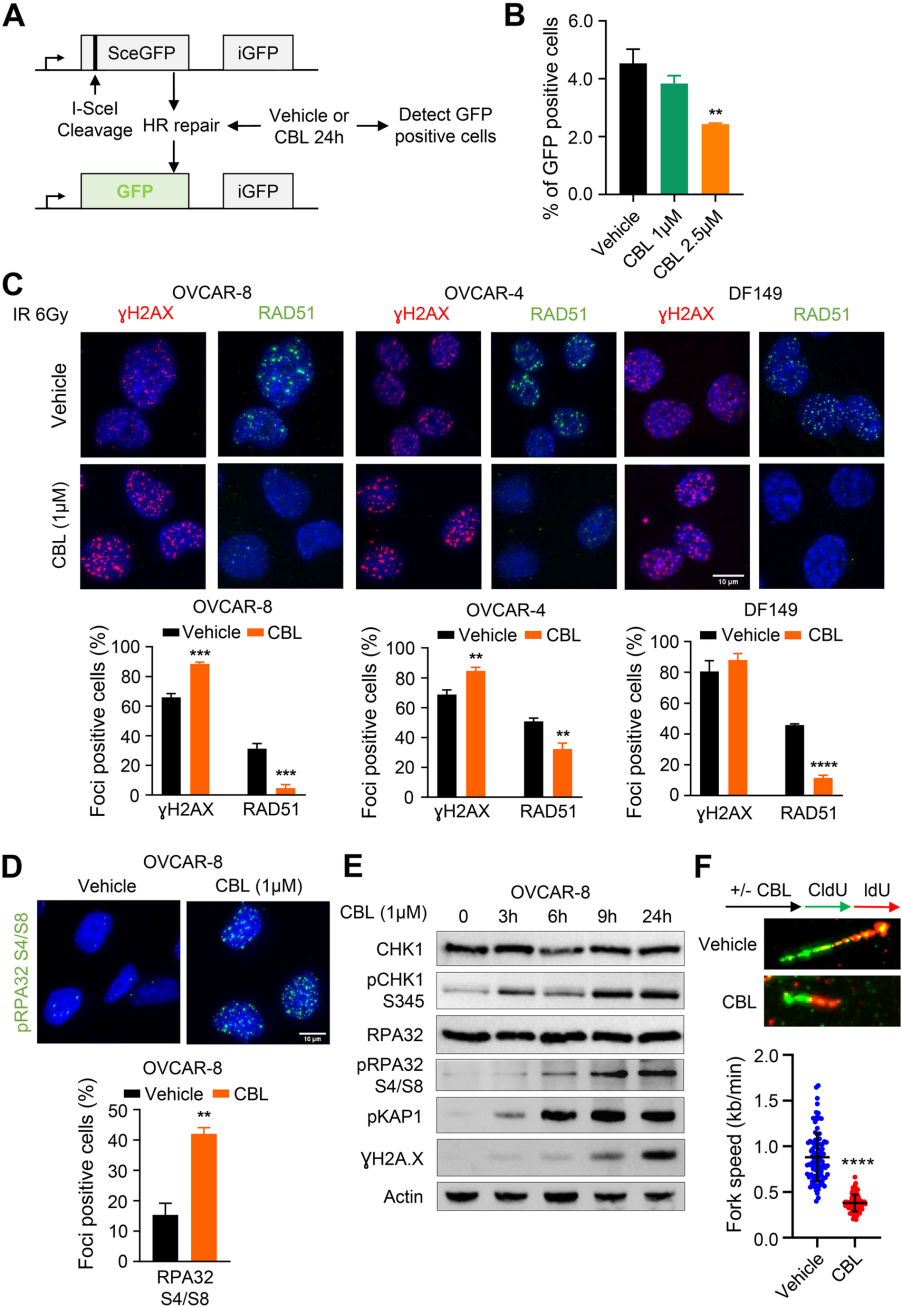
CBL0137 represses IR-induced RAD51 foci recruitment. **A** Schematic of HR reporter assay. Sce is a modified GFP gene, which contains I-Sce site and iGFP is internal GFP fragment that allows repair of breaks by HR resulting in functional GFP gene. **B** Quantification of the percentage of GFP positive cells after CBL0137 treatment. Statistical analysis was performed by One-way ANOVA (** for *P* ≤ 0.01). **C** Immunofluorescent visualization and quantification of RAD51 and ɣH2A.X foci in OVCAR-8, OVCAR-4, and DF149 cells. Cells were treated with vehicle or 1 μM CBL0137 for 3 h prior to 6 h incubation after 6-Gy irradiation. Blue color represented DAPI, green color was RAD51, and red color displayed ɣH2A.X foci. A cell with more than 5 distinct RAD51 foci or 10 distinct ɣH2A.X foci in the nucleus was positive. Data represented percentage of positive cells mean ± SEM (*n* = 3). Unpaired Student’s t-test was employed for statistical significance. **D** Immunofluorescent visualization and quantification of RPA32 foci in OVCAR-8 cells treated with vehicle or 1 μM CBL0137 for 6 h. Blue color represented DAPI and green color represented pRPA32 S4/S8. Data represented percentage of positive cells mean ± SEM (*n* = 3). Unpaired Student’s t-test was employed for statistical significance. **E** Western blot analysis of OVCAR-8 cells treated with 1 μM of CBL0137 for indicated time. Actin was applied as loading control. **F** DNA fiber assay of OVCAR-8 cells pre-treated with 1 μM of CBL0137 for 3 h, washed and labelled with CldU and IdU as shown in the schematic illustration (Upper panel). Representative images of DNA fibers from untreated and CBL0137-pretreated cells (Middle panel). Replication fork speed was calculated by length of track/time of IdU pulse (Lower panel). Representative experiment data was shown from two independent fiber assays repeated. Data represents fork speed mean ± SD (*n* = 100). Statistical significance was determined by Unpaired Student’s t-tests (** for *P* ≤ 0.01, *** for *P* ≤ 0.001, and **** for *P* ≤ 0.0001)

To demonstrate that CBL0137 directly compromises HR repair in HGSC cells, we utilized HR-proficient OVCAR-8 cells engineered to express widely used DR-GFP reporter wherein GFP expression is observed only upon proper HR repair of I-SceI endonuclease-induced DNA double-strand breaks (Fig. 4A) [47]. CBL0137 treatment significantly disrupted HR activity in OVCAR-8 cells (Fig. 4B). Next, we assessed functional defect in HR-repair using DNA damage-induced RAD51 nuclear foci formation, as a marker for the HR competency [48]. HR-proficient OVCAR-8, OVCAR-4 and patient-derived DF149 cells were pre-treated with CBL0137 (1 μM) for 3 h followed by 6-Gy irradiation (IR) exposure, then allowed to recover for 6 h. Immunofluorescence analysis was used to mark sites of IR-induced DNA double-strand breaks induction (_Ƴ_H2AX foci), and RAD51 nuclear foci formation, in all three cell lines. CBL0137 pre-treatment significantly reduced IR-induced RAD51 nuclear foci formation in OVCAR-8, OVCAR-4 and DF149 cells (Fig. 4C). There was no reduction in RAD51 protein levels after CBL0137 treatment (Additional file 2, Fig. S3A). CBL0137 also significantly increased IR-induced DNA damage (_Ƴ_H2AX foci-positive cells) induction in both OVCAR-8 and OVCAR-4 cells (Fig. 4C). In contrast, CBL0137 treatment had no significant effect on IR-induced RAD51 foci formation in SSRP1-low HR-proficient PEO4 cells (Additional file 2, Fig. S3B). HR-deficient PEO1 cells with mutation in *BRCA2* gene were used as a control for defective DNA damage-induced RAD51 foci formation (Additional file 2, Fig. S3B).

We next examined if the defect in HR was due to a defect in resection of IR-induced double-strand breaks. We hence analysed the effect of CBL0137 on phospho-RPA32 S4/S8 foci formation. CBL0137 treatment itself induced significant RPA32 foci formation in OVCAR-8 (Fig. 4D) and OVCAR-4 (Additional file 2, Fig. S4A) cells, suggesting that CBL0137 induces replication stress in HGSC cells. Moreover, CBL0137 treatment also resulted in marked increase in phospho-RPA32 S4/S8 and phospho-CHK1 S345 levels and other DNA damage response markers in OVCAR-8 (Fig. 4E) and OVCAR-4 (Additional file 2, Fig. S4B) cells in a time-dependent manner indicating progressive replication stress in treated cells. We then performed DNA fiber assay to analyze the effect of CBL0137 on replication fork progression in OVCAR-8 and OVCAR-4 cells. CBL0137 treatment significantly reduced fork speed as the mean fork speed in control DMSO-treated OVCAR-8 cells is around 0.88 kb/min compared to that of CBL-treated which is 0.38 kb/min (Fig. 4F). Similar to OVCAR-8 cells, CBL0137 treatment significantly reduced the mean fork speed in control DMSO-treated OVCAR-4 cells from 0.63 kb/min to that of 0.25 kb/min in CBL-treated OVCAR-4 cells (Additional file 2, Fig. S4C). Taken together, our data indicated that CBL0137 impairs DNA repair via HR pathway in *BRCA1/2* proficient cells through downregulation of MYC and further induces replication stress via impacting replication fork stability.

### CBL0137 exerts synergistic anti-tumor activity with PARPi in HGSCs

Defective HR is known to induce synthetic lethality with PARPi, however, PARPi have limited efficacy on HR proficient cells which account for approximately 50% of HGSC patients [49]. PARPi are currently evaluated in different combination therapy settings. Nuclear RAD51 foci formation is a functional biomarker of HR repair, the absence of which has consistently predicted sensitivity and objective response to PARPi in patient tumors [50]. Moreover, replication fork destabilization also leads to PARPi sensitivity in cancer cells. Based on the ability of CBL0137 to compromise HR by suppressing RAD51 foci formation and its ability to cause replication stress, we assessed the effect of combination treatment of CBL0137 with Olaparib and Rucaparib, clinically approved PARPi, on the growth of HGSCs cell lines.

**Fig. 5:**
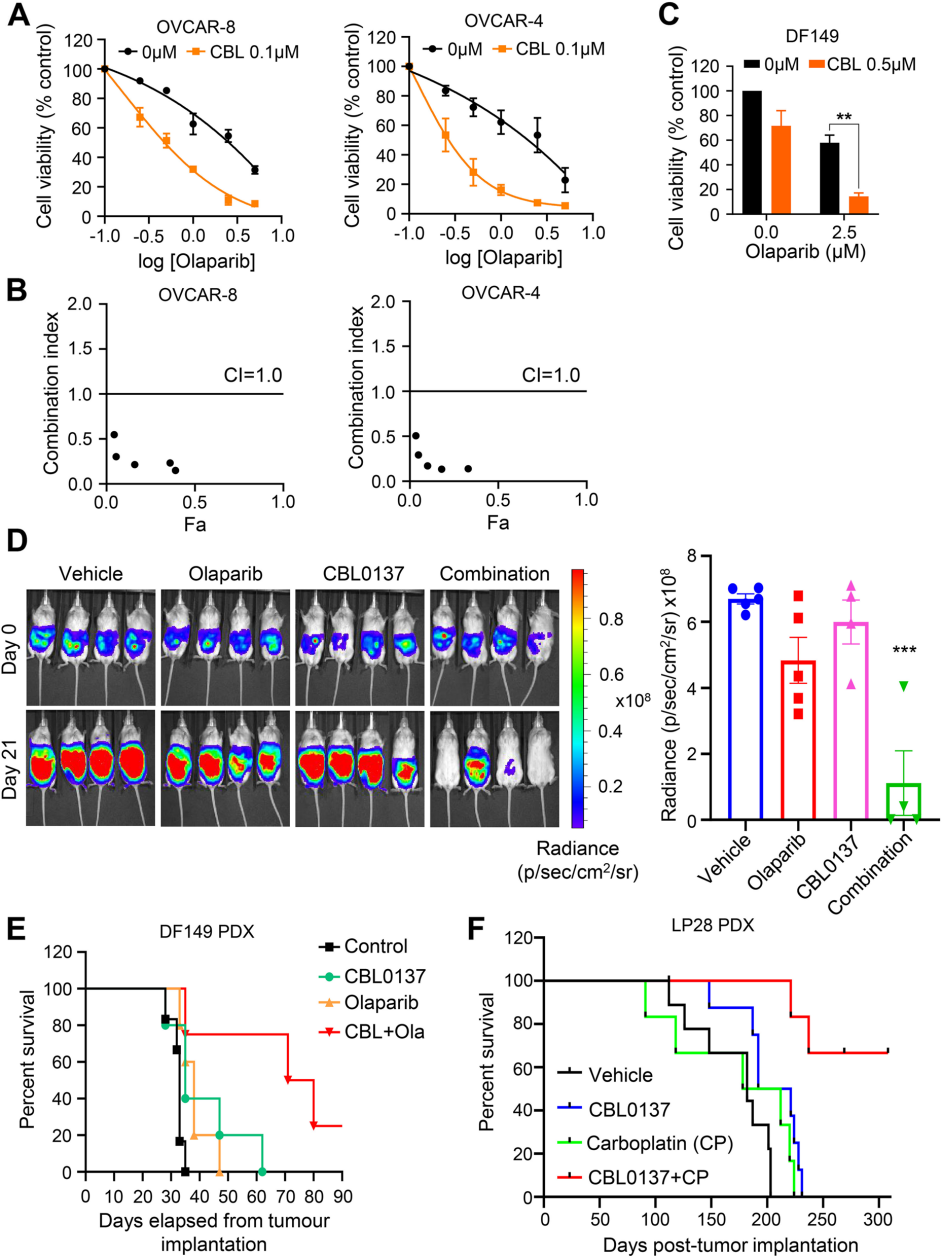
CBL0137 exerts a synergistic anti-cancer activity with PARPi in HGSCs. **A** OVCAR-8 and OVCAR-4 cells were seeded (3000 cells/well) in the 6-wells plate and treated with or without 0.1 μM CBL0137 for 24 h prior to the treatment with Olaparib (0-5 μM) for subsequent 14 days. **B** Combination index (CI) was calculated using CompuSyn software. Synergistic effects, additive effects, and antagonistic effects are defined by CI values of < 1, =1, and > 1, respectively. **C** DF149 cells were seeded (50,000 cells/well) in the 6-wells plate and treated with or without 0.5 μM CBL0137 for 24 h prior to the treatment with Olaparib (0 or 2.5 μM) for subsequent 14 days. The cells were then stained by crystal violet. Quantification of the colonies of combination treatments with Olaparib in all cell lines were measured by reading crystal violet absorbance after destaining. **D** NRG mice bearing HGSC patient-derived luciferase-tagged DF149 tumor cells were treated with vehicle, CBL0137, Olaparib, or combination for 2 weeks (*n* = 5 mice in control and olaparib groups, *n* = 4 mice in CBL0137 and combination groups as one mouse from each group was euthanized due to CBL0137-induced tail necrosis before the endpoint). For combination group, mice were treated with CBL0137 24 h prior to Olaparib treatment. Tumor growth was monitored by bioluminescence imaging and survival of tumor-bearing mice was monitored. Representative images and quantification analysis of tumor burden on day 0 and day 21 is shown. **E** The Kaplan-Meier survival analysis of mice from each treatment group was performed. Log-rank test was applied for statistical significance analysis (*P* = 0.0021). **F** The Kaplan-Meier survival analysis on mice bearing LP28 HGSC PDX following treatment with CBL0137 or carboplatin alone and in combination. Log-rank test was applied for statistical significance analysis (*P* = 0.0001)

We tested the growth inhibitory activity of CBL0137 in combination with PARPi Olaparib in a long-term clonogenic assay using SSRP1-high OVCAR-8, OVCAR-4, PEO1, and SSRP1-low PEO4 cells. CBL0137 pre-treatment (24 h) significantly decreased Olaparib IC_50_ in HR-proficient and SSRP1-high OVCAR-8 and OVCAR-4 cells (Fig. 5A, Additional file 2 – Fig. S5A). CompuSyn analysis revealed the strong synergistic activity of CBL0137 combined with Olaparib in OVCAR-8 and OVCAR-4 cells (Fig. 5B). Similarly, CBL0137 also demonstrated synergy with Olaparib in HR-proficient patient-derived DF149 cells in vitro (Fig. 5C, Additional file 2 – Fig. S5C). Similar observations were made using HR-deficient SSRP1-high PEO1 cells (Additional file 2, Fig. S5A, S5B), suggesting additional fork-destabilization effect of CBL0137 might also account for PARPi sensitivity. However, CBL0137 pre-treatment (24 h) failed to sensitize HR-proficient but SSRP1-low PEO4 cells to Olaparib (Additional file 2, Fig. S5A, S5B), suggesting that SSRP1 may serve as a biomarker for CBL0137 activity as a monotherapy and also in combination with Olaparib. Similar to Olaparib, CBL0137 pre-treatment (24 h) significantly sensitized HR-proficient and SSRP1-high OVCAR-8 and OVCAR-4 cells as well as HR-deficient SSRP1-high PEO1 cells to another PARPi, Rucaparib (Additional file 2, Fig. S6A, B), suggesting that observed synergistic anti-cancer activity of CBL0137 in SSRP1-high HGSCs is relevant to a number of clinically approved PARPi.

Since CBL0137 sensitized SSRP-high HR-proficient HGSC cells to PARPi, we examined if CBL0137 and Olaparib combination therapy induces accumulative DNA damage compared to CBL0137 or Olaparib monotherapy. Compared to single-agent, the combination of PARPi and CBL0137 produced more DNA damage and replication stress as evidenced by increased induction of classical DNA damage markers phospho-KAP1 and phospho-_Ƴ_H2AX and replication stress marker phospho-RPA32 S4/S8 in SSRP1-high HR-proficient OVCAR-8 and OVCAR-4 cells (Additional file 2, Fig. S5D). These data suggested that CBL0137 and Olaparib combination treatment induces DNA damage and replication stress in HGSC cells which may contribute to its synergistic anti-cancer activity.

We next evaluated the synergistic anti-tumor activity of CBL0137 and Olaparib in vivo using the HR-proficient SSRP1-high DF149 HGSC PDX [30]. DF149 tumor-bearing female NRG mice were treated with half maximum-tolerated dose (MTD) of CBL0137 (30 mg/kg) and Olaparib (50 mg/kg), alone or in combination, for 2 weeks and tumor growth was monitored using bioluminescence imaging. Our results showed that both CBL0137 and Olaparib monotherapy had negligible effect on tumor growth of DF149 PDX because mice were treated with only half-MTD dose that is not sufficient to reduce tumor burden as a monotherapy. However, the combination treatment was significantly effective in reducing tumor burden by approximately 6-fold (Fig. 5D). This correlated with significant improvement of survival of mice treated with combination treatment to 75.5 days from 33 days in the vehicle-treated group (Log-rank *P* < 0.01, Fig. 5E). Similar observations were also made using syngeneic HGSC model, ID8 *p53*^*−/−*^ *BRCA* wild-type [29] wherein the CBL0137 and Olaparib combination significantly prolonged the median survival to 76 days compared to 53.5 days in vehicle-treated mice, 55 days in CBL0137 alone, and 53.5 days in Olaparib alone (Additional file 2, Fig. S6C). The combination was well-tolerated as no significant weight loss was observed. Thus, our data indicated that CBL0137 sensitizes SSRP1-high HGSCs to PARPi in pre-clinical models of HGSCs in vivo.

Since CBL0137 induces HR deficiency, we examined if CBL0137 enhances the efficacy of DNA damaging platinum-based chemotherapy. We engrafted HGSC PDX model LP28 intraperitoneally into 6-week-old female NSG mice and treated with CBL0137 and carboplatin, both as a single agent and in combination. CBL0137 and carboplatin combination significantly prolonged survival of LP28 tumor-bearing mice (Fig. 5F). The median survival was observed to be 182 days in vehicle-treated mice, 206.5 days in CBL0137-treated mice, 195 days in carboplatin-treated mice, and > 300 days in combination treatment group. Hence, CBL0137 may increase the efficacy of carboplatin in HGSC patients that have developed resistance to cisplatin/carboplatin.

## Discussion

The identification of effective targeted options for patients with HR-proficiency (HRP) HGSC is urgently required and is a high priority given the poor prognosis and limited to no benefit with PARPi. Herein, we report a novel targeted strategy using CBL0137, a small molecule inhibitor known to trap FACT complex components onto chromatin and have demonstrated efficacy in HRP HGSC cell lines in vitro and in vivo as a single agent and combined with a PARPi. The FACT complex components SSRP1 and SPT16 are normally expressed during embryonic development and in primitive tissues and their expression is lost during tissue differentiation and in mature tissues [51, 52]. Several studies have reported upregulation of FACT complex components in multiple cancers and FACT activation is often associated with more aggressive disease and worse outcomes [25, 28, 53, 54]. FACT complex components, especially SSRP1, play important roles in regulating the cell cycle, gene transcription and DNA repair in cancer cells [14, 55]. Hence, SSRP1 represents a potential therapeutic target in multiple human cancers and several studies have reported that pharmacological inhibition of the FACT complex using CBL0137 exerts significant anti-tumor activity [24, 28, 54, 56]. Since SSRP1 expression is high in HGSCs, in this study we evaluated the anti-cancer activity of CBL0137 in a range of pre-clinical models of HGSC including cell lines, patient-derived tumor cells ex vivo, syngeneic murine models and PDXs. In line with previous studies [20, 26, 28], our data indicated that CBL0137 traps SSRP1 onto chromatin and induces apoptosis in SSRP1-high HGSC cells in vitro and exerts significant growth inhibitory activity in vivo*.* Consequently SSRP1-low expressing HGSCs cell lines or tumor cells or SSRP1-inhibition in SSRP1-high HGSCs lines significantly reduced sensitivity to CBL0137. Thus, SSRP1 expression should be taken into consideration for treatment of HGSCs with CBL0137.

FACT promotes DNA damage repair in cancer cells in response to treatment with DNA-damaging chemotherapy [57]. In response to DNA single strand breaks, SSRP1 is recruited to the damage site in a PARP1-dependent manner where it interacts with DNA repair protein XRCC1 [14]. Additionally, CBL0137-induced SSRP1 chromatin trapping has been shown to downregulate DNA repair pathway in MYC-high medulloblastoma cells [54]. In line with these studies, our RNA-seq results demonstrated that CBL0137 treatment downregulated DNA repair pathways in SSRP1-high patient-derived HGSC cells DF149. Furthermore, CBL0137 pre-treatment also reduced RAD51 foci formation, a functional readout of HR repair, in response to ionizing radiation in SSRP1-high HGSC cells. Indeed, in SSRP1-low cells, CBL0137 treatment does not significantly decrease expression of MYC, HR genes and HR repair. Hence, CBL0137-induced SSRP1 trapping impairs DNA repair machinery in SSRP1-high HGSC cells and may sensitize them to DNA damaging therapy including platinum- chemotherapy and PARPi.

Herein we showed that CBL0137 and carboplatin combination treatment significantly prolonged the survival of mice bearing cisplatin-resistant SSRP1-high LP28 HGSC patient-derived tumors. These results are in line with previous studies where CBL0137 treatment enhanced the efficacy of platinum-based chemotherapy in medulloblastoma and non-small cell lung carcinomas [24, 58]. Hence, using CBL0137 in combination with carboplatin, a front-line treatment for HGSCs, may improve treatment outcomes in SSRP1-high ovarian cancer patients and deserves investigation.

PARPi including Olaparib, Rucaparib, and Niraparib demonstrate significant clinical benefit as maintenance therapy following response to platinum-based chemotherapy in ovarian cancer patients with *BRCA1/2* mutation and/or HR-deficiency in both the 1st line and recurrent setting [6, 8–10]. Extending the efficacy of PARPi to *BRCA1/2* wild-type HR proficient ovarian cancer patients is an unmet clinical need. Additionally, acquisition of HR-proficiency through reversion mutations is the dominant mechanism of PARPi-resistance in *BRCA1/2* mutated HGSC patients [59]. Recently, BRD4 inhibitor JQ1 and CDK4/6 inhibitor palbociclib have been shown to inhibit HR and sensitize *BRCA1/2* wild-type ovarian cancers and other cancers to PARPi [60–62]. Notably, our data demonstrates that CBL0137 induces HR deficiency in SSRP1-high ovarian cancer cells. Consistent with this, CBL0137 and Olaparib combination treatment exerts a significant anti-tumor activity in HR-proficient DF149 patient-derived tumor xenografts and a HR-proficient syngeneic murine ID8 HGSC model, resulting in a significant prolongation of the overall survival of tumor bearing mice. These findings provide a novel clinically applicable therapeutic strategy using CBL0137 to sensitize HR-proficient SSRP1-high ovarian cancer patients to PARP inhibitors thereby broadening the scope of PARPi irrespective of *BRCA1/2* mutation status and HR status. Although we showed that CBL0137 sensitized HR-proficient HGSCs to PARPi, the efficacy of CBL0137 in overcoming PARPi resistance in *BRCA1/2* mutated ovarian cancer patients is yet to be examined and is an important next step.

## Conclusion

Our results suggest that CBL0137 holds a potential as a novel targeted therapy for patients with SSRP1-high HGSC. Our results indicate that CBL0137 can be combined with PARPi for the treatment of HR-proficient SSRP1-high grade HGSC and deserves clinical investigation. Notably, SSRP1 is overexpressed in number of major cancer types, thus, our discovery could have far-reaching implications for broadening the scope of PARPi.

## Supplementary Information

**Additional file 1: **Supplementary methods.

**Additional file 2: **Supplementary figs. S1 – S5.

**Additional file 3: **Supplementary figs. T1 – T8.

## Data Availability

The datasets used and/or analysed during the current study are available from the corresponding author on reasonable request. The materials used in this study are available from the corresponding author upon request. The RNA sequencing dataset supporting the conclusions of this article is available in the [The European Genome-phenome Archive (EGA)] repository, [EGAS00001006662]. The RNA sequencing analysed data supporting the conclusions of this article are included within the article and its additional files.

## Abbreviations

CI: Combination Index
CT: Carboplatin
EOC: Epithelial Ovarian Cancer
FACS: Flow Cytometry
FACT: FAcilitates Chromatin Transcription
FBS: Fetal Bovine Serum
H2A.X: H2A histone family member X
HGSC: High-Grade Serous Ovarian Cancer
HR: Homologous Recombination
HRD: Homologous Recombination Deficiency
HRP: Homologous Recombination Proficiency
IR: Irradiation
MTD: Maximum-Tolerated Dose
OEC: Ovarian Epithelial Cells
PARP: Poly (ADP-ribose) Polymerase
PARP1: Poly (ADP-ribose) Polymerase 1
PARPi: Poly (ADP-ribose) Polymerase inhibitors
PDX: Patient-Derived Xenograft
RNA-Seq: RNA sequencing
RT-qPCR: Reverse Transcription quantitative PCR
siRNA: Small Interfering RNA
SSRP1: Structure Specific Recognition Protein 1
XRCC1: X-Ray Repair Cross Complementing 1
XRCC2: X-Ray Repair Cross Complementing 2
