# Supplementary material for: CBL0137 impairs homologous recombination repair and sensitizes high-grade serous ovarian carcinoma to PARP inhibitors
Source: J Exp Clin Cancer Res. 2022 Dec 21;41:355. doi: 10.1186/s13046-022-02570-4 (PMC9769062; doi:10.1186/s13046-022-02570-4)
Supplement: Supplementary file 1 — Additional file 1: Supplementary methods. [file 13046_2022_2570_MOESM1_ESM.docx]

**SUPPLEMENTARY METHODS:**

**Cell proliferation assays:**

HGSC cell lines and patient-derived tumor cells were treated with CBL0137 (0-2.5 µM) for 96 h in 48-well plates. Cell viability was measured by the CellTiter 96® Aqueous One Solution Cell Proliferation Assay (MTS) (Promega) per the manufacturer’s guidelines.

**siRNA transfection:**

Cells were transfected with 20 nM of non-specific scramble or SSRP1-specific small interfering RNAs (siRNAs) using Lipofectamine RNAi MAX (Invitrogen Cat #: 13778030) per manufacturer’s instructions.

**Colony formation assays:**

HGSC cells (3,000) were seeded overnight in 6-well plates then treated with or without CBL0137 (0.1 µM) for 24 hours prior to the treatment with Olaparib (0-5 µM) for subsequent 14 days for colony formation. The colonies were fixed with 0.05% crystal violet for 30 mins. The quantification of crystal violet intensity was measured after destaining colonies by Sorenson’s buffer (0.1 M sodium citrate in 50% ethanol, pH 4.2) by a PowerWave HT Microplate Spectrophometer (BioTek, USA) at 590 nM absorbance.

**Cell cycle analysis**

OVCAR-8, OVCAR-4, PEO1 and PEO4 cells treated with CBL0137 (0-2.5 µM) were harvested, washed in PBS with 5% FBS and fixed in ice-cold ethanol for 24 h. Cells were stained in 1 mg/mL of propidium iodide (Sigma Aldrich) and 15 mg/mL RNase A at 37°C. Data acquisition was performed by FACS canto II flow cytometry (BD Biosciences, Mountain View, CA). Cell cycle was analyzed using MODFIT LT4.0 software Verity (Software House, Topsham, ME, USA).

**Annexin V staining**

For apoptosis determination, cells were treated with CBL0137 (0-2.5 µM) for 48 h. Then the cells were harvested, washed, and stained with Annexin V-FITC conjugate (Invitrogen Cat #: A13201). Fluorescence was detected instantly on a BD FACS Canto II flow cytometry. The analysis was performed using FlowJo V 10 (Tree Star, Ashland, Oregon, USA).

**Immunoblotting**

HGSC and PDX cells were treated with CBL0137 (0-2.5 µM) for 24 h. Protein was extracted using Urea buffer. Immunoblotting was performed as described previously (1) with antibodies listed in Table S2. The Super Signal chemiluminescent ECL-plus (Amersham) was applied for the target protein detection.

**Reverse transcription-quantitative PCR**

Reverse transcription quantitative PCR (RT-qPCR) was performed on a CFX384 Touch™ Real-Time PCR Detection System (Bio-Rad) using SYBR™ Green PCR Master Mix (Applied Biosystems, Cat #: 4309155) as described previously (2). The list of primers used is listed in Table S3.

**References:**

1. Makhale A, Nanayakkara D, Raninga P, Khanna KK, Kalimutho M. CX-5461 Enhances the Efficacy of APR-246 via Induction of DNA Damage and Replication Stress in Triple-Negative Breast Cancer. International journal of molecular sciences **2021**;22

2. Raninga PV, Lee A, Sinha D, Dong LF, Datta KK, Lu X*, et al.* Marizomib suppresses triple-negative breast cancer via proteasome and oxidative phosphorylation inhibition. Theranostics **2020**;10:5259-75
