# Supplementary material for: CBL0137 impairs homologous recombination repair and sensitizes high-grade serous ovarian carcinoma to PARP inhibitors
Source: J Exp Clin Cancer Res. 2022 Dec 21;41:355. doi: 10.1186/s13046-022-02570-4 (PMC9769062; doi:10.1186/s13046-022-02570-4)
Supplement: Supplementary file 2 — Additional file 2: Supplementary figs. S1 – S5. [file 13046_2022_2570_MOESM2_ESM.docx]

**SUPPLEMENTARY FIGURES**

**Figure S1:**

***
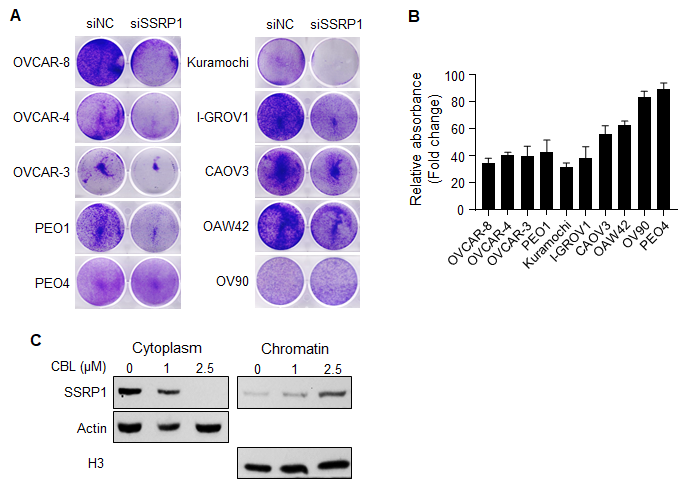
***

***Figure S1.*** ***SSRP1 knockdown reduces HGSC cell viability:***

***(A)*** *A panel of HGSC lines were transfected with either scramble or SSRP1-specific siRNA for 48 h, followed by the culture in fresh media for 14 days. The cells were then stained with crystal violet and representative images were shown.* ***(B).*** *Quantification data was obtained by measuring crystal violet absorbance.* *Data displayed as relative absorbance mean ± SEM (n=3).* ***(C)*** *OVCAR-8 cells were treated with CBL0137 (0-2.5 µM) for 24 h and SSRP1 protein levels were analyzed in the cytoplasmic and chromatin fractions. Representative Immunoblot images of three independent experiments are shown. Actin or Histone H3 were used as loading controls.*


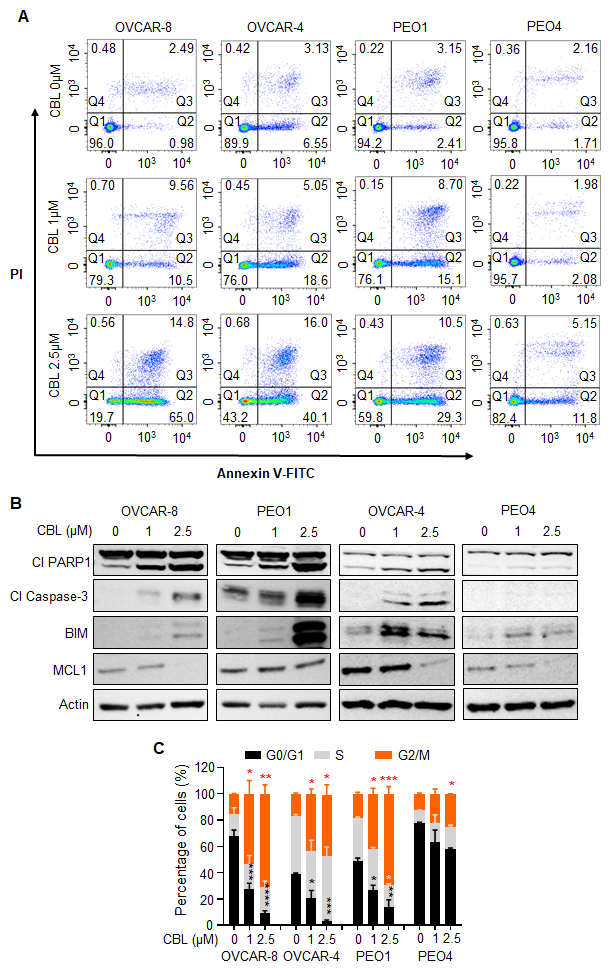


***Figure S2: CBL0137 induces apoptosis in SSRP1-high HGSC cells.***

*(****A)*** *Annexin V FITC/PI flow cytometry analysis of OVCAR-8, OVCAR-4, PEO1, and PEO4 cells treated with 0, 1, and 2.5µM of CBL0137 for 48 h. Representative images were shown from three biological repeats.* ***(B)*** *The protein expression of apoptosis markers including cleaved PARP1, cleaved caspase-3, BIM, and MCL1 were evaluated after CBL0137 treatment (24 h, 0-2.5 µM) in a panel of HGSC cell lines. Actin was used as a loading control.* ***(C)*** *OVCAR-8, OVCAR-4, PEO1, and PEO4 cells were treated with CBL0137 (24 h, 0-2.5 µM) and cell cycle analysis was performed using propidium iodide staining and flow cytometry. The quantitative measurement represents the cell cycle phase of each cell line. Values indicate percentage of cells mean ± SEM (n=3). One-way ANOVA analysis was employed for statistical significance (* for P ≤ 0.05, ** for P ≤ 0.01, *** for P ≤ 0.001, and **** for P ≤ 0.0001).*

***
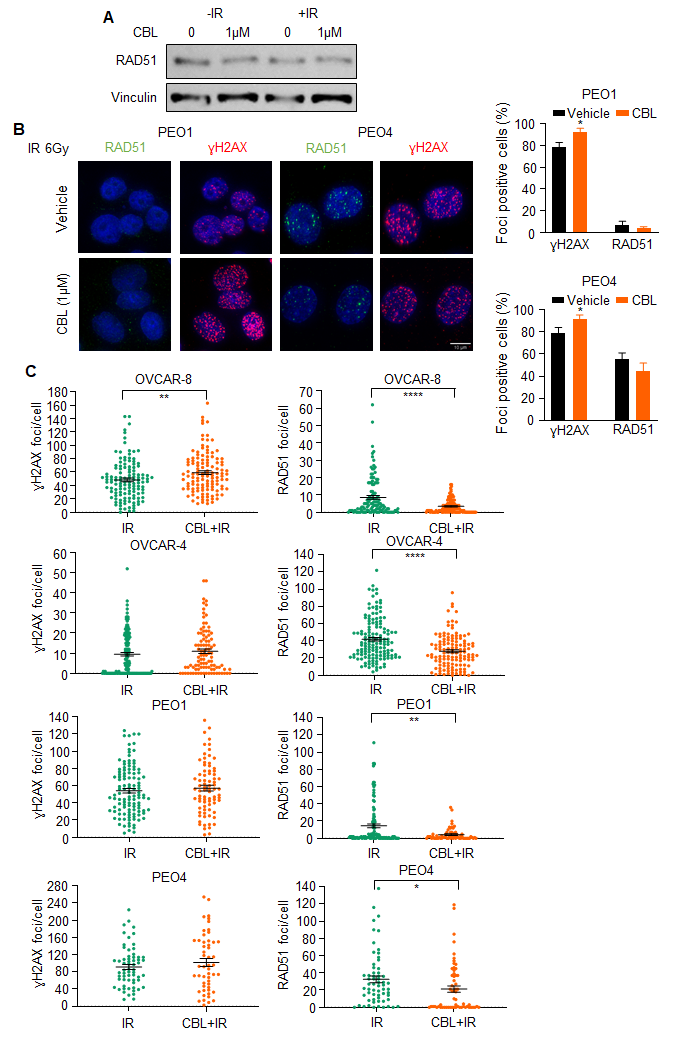
***

***Figure S3: CBL0137 impairs homologous recombination repair in HGSC.***

***(A)*** *The protein expression of RAD51 was evaluated after CBL0137 treatment (8 h, 0 or1 µM) with or without 6-Gy IR challenge in OVCAR-8 cells. Vinculin was used as a loading control.* ***(B)*** *Immunofluorescent visualization and quantification of RAD51 and ɣH2A.X foci in PEO1, and PEO4 cells. Cells were treated with vehicle or 1 µM CBL0137 for 2 h prior to 6 h incubation after 6Gy irradiation. Blue color represented DAPI, green color was RAD51, and red color displayed ɣH2A.X foci. A cell with more than 5 distinct RAD51 foci or 10 distinct ɣH2A.X foci in the nucleus was considered to be positive. Data represented percentage of positive cells mean ± SEM (n=3). Unpaired Student’s t-test was employed for statistical significance.* ***(C)*** *Quantification of RAD51 and ɣH2A.X foci per cell in OVCAR-8, OVCAR-4, PEO1, and PEO4 cells was analysed by QuPath software. Unpaired Student’s t-test was employed for statistical significance (* for P ≤ 0.05, ** for P ≤ 0.01, and **** for P ≤ 0.0001).*

*
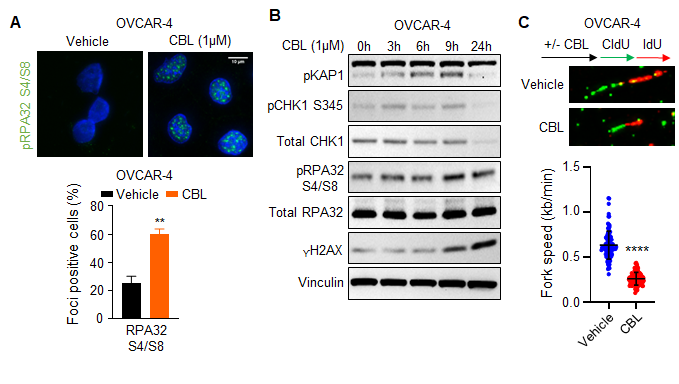
*

***Figure S4: CBL0137 induces replication stress and reduces replication fork progression in HGSC.***

***(A)*** *Immunofluorescent visualization and quantification of RPA32 foci in OVCAR-4 cells treated with vehicle or 1 µM CBL0137 for 6 h. Blue color represented DAPI and green color represented pRPA32 S4/S8. Data represented percentage of positive cells mean ± SEM (n=3). Unpaired Student’s t-test was employed for statistical significance.* ***(B)*** *Western blot analysis of OVCAR-4 cells treated with 1 µM of CBL0137 for indicated time. Actin* *was applied as loading control.* ***(C)*** *DNA fiber assay of OVCAR-4 cells pre-treated with 1 µM of CBL0137 for 3 h, washed and labelled with CldU and IdU as shown in the schematic illustration (Upper panel). Representative images of DNA fibers from untreated and CBL0137-pretreated cells (Middle panel). Replication fork speed was calculated by length of track/time of IdU pulse (Lower panel). Representative experiment data was shown from two independent fiber assays repeated. Data represents fork speed mean ± SD (n=100). Statistical significance was determined by Unpaired Student’s t-tests (** for P ≤ 0.01, *** for P ≤ 0.001, and **** for P ≤ 0.0001).*

*
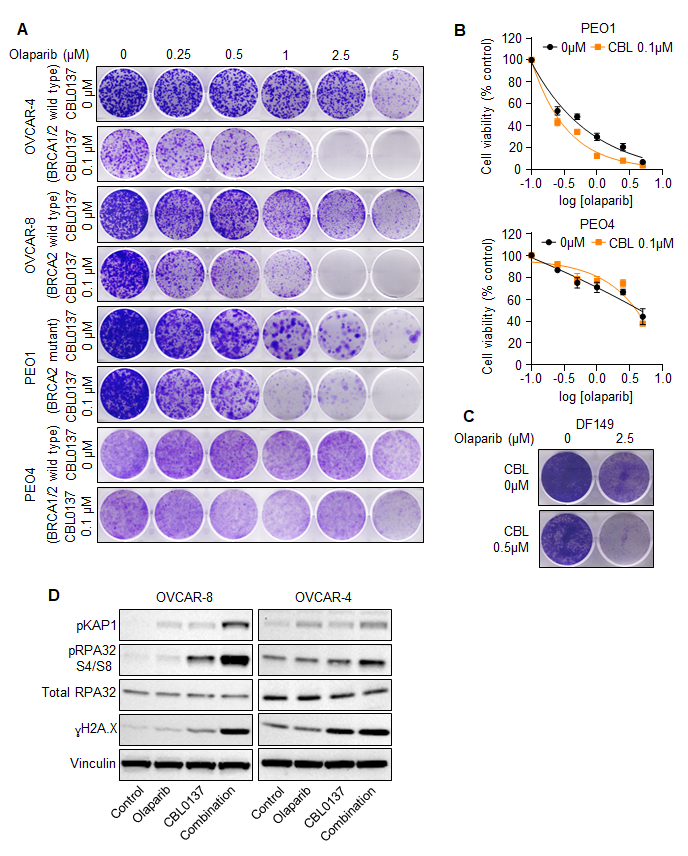
*

***Figure S5:*** ***CBL0137 exerts a synergistic anti-cancer activity with PARPi in HGSCs.***

***(A)*** *OVCAR-4, OVCAR-8, PEO1, and PEO4 cells were seeded 3000 cells/well in the 6-well plates and then treated with or without 0.1µM CBL0137 for 24 h, following treatment with Olaparib (0-5µM) for 14 days. The cells were then stained by crystal violet staining. Representative images are shown (n=3).* ***(B)*** *Quantification of cell viability of PEO1 and PEO4 cells treated with CBL0137 and Olaparib combination.* ***(C)*** *DF149 cells were seeded 50,000 cells/well in the 6-well plates and then treated with or without 0.5 µM CBL0137 for 24 h, following with Olaparib (0 or 2.5 µM) for 14 days. The cells were then stained by crystal violet staining. Representative images are shown (n=3).* ***(D)*** *OVCAR-8 and OVCAR-4 cells were pre-treated with CBL0137 (1 µM) 3 h and then subsequently treated with Olaparib (5 µM) for 24 h. Protein levels of phospho-KAP1, phospho-RPA32-S4/S8, total RPA32, and _Ƴ_H2AX were analysed by Western blotting. Vinculin was used as a loading control.*

***
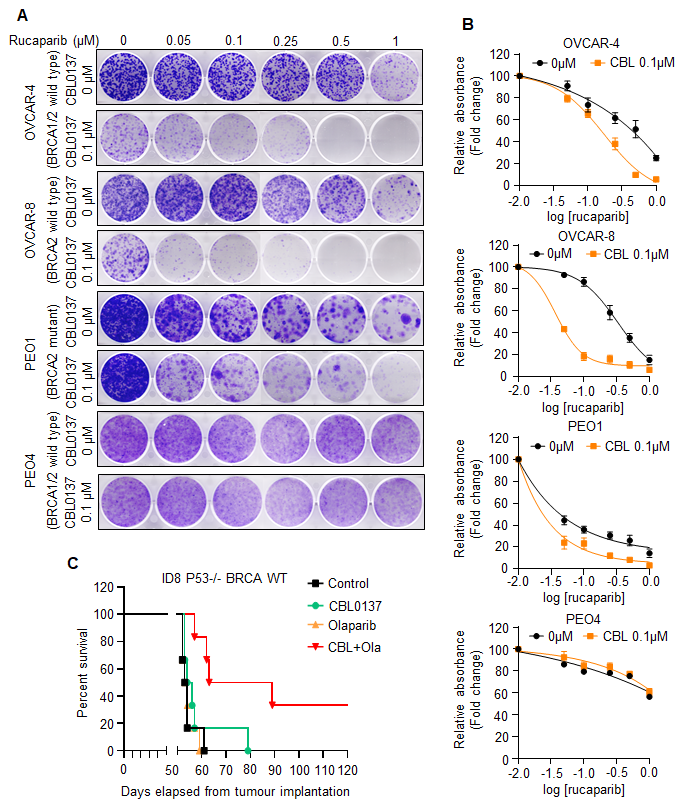
***

***Figure S6: CBL0137 exerts a synergistic anti-cancer activity with another PARP inhibitor, Rucaparib.***

***(A, B)*** *OVCAR-4, OVCAR-8, PEO1, and PEO4 cells were treated with CBL0137 (0.1 µM) for 24 h, followed by treatment with Rucaparib (0-1 µM) for 14 days. Cells were stained with crystal violet. (A) Representative images are shown (n=3). (B) Quantification of the crystal violet staining for each cell line is presented.* ***(C)*** *C57BL/6 mice bearing ID8 P53-/- BRCA WT murine HGSC tumours were treated with vehicle, CBL0137 (30 mg/kg, once a week, i.v), Olaparib (50 mg/kg, Mon-Fri, i.p), or combination for 2 weeks (n=6 mice in each group). The Kaplan-Meier survival analysis was performed.*
